# Critical care management of systemic mastocytosis: when every wasp is a killer bee

**DOI:** 10.1186/s13054-015-0956-z

**Published:** 2015-06-03

**Authors:** Hinke Y. van der Weide, David J. van Westerloo, Walter M. van den Bergh

**Affiliations:** Department of Critical Care, University Medical Center Groningen, University of Groningen, Hanzeplein 1, 9700 RB Groningen, The Netherlands; Department of Intensive Care, Leiden University Medical Center, Albinusdreef 2, 2333 ZA Leiden, The Netherlands

## Abstract

Since the critical care physician will most likely be involved in a life-threatening expression of systemic mastocytosis, recognition of this disease is of utmost importance in the critical care management of these patients. Mastocytosis is a severely under-recognized disease because it typically occurs secondary to another condition and thus may occur more frequently than assumed. In this article, we will review the current knowledge on the treatment of mastocytosis crises with an emphasis on critical care management. Mastocytosis is characterized by the clonal proliferation and accumulation of mast cells in different tissues. Mast cell mediators contain a wide range of biologically active substances that may lead to itching and hives but may ultimately lead to anaphylactic shock caused by the release of histamine and other mediators from mast cells. The mainstay of therapy is the avoidance of potential triggers of mast cell degranulation and, if unsuccessful, blocking the cascade of mast cell mediators. The critical care physician should be well aware of the special precautions which should be kept in mind throughout the management of a mastocytosis crisis to avoid massive mast cell degranulation. Histamine-releasing drugs and certain physical triggers like temperature change should be avoided.

## Introduction

Mastocytosis is characterized by the clonal proliferation and accumulation of mast cells in different tissues, with a preferential localization in the skin and bone marrow. The clinical presentation of mastocytosis is heterogeneous, ranging from skin-limited to a more aggressive systemic variant [1]. Mast cells produce and release a large number of different mediators, which are either released continuously or stored in granules and released after stimulation (Table 1). Signs and symptoms are caused by release of these mast cell mediators or local accumulation of mast cells [2, 3]. The disease may be undiagnosed until patients are exposed to elicitors of mast cell degranulation leading to severe anaphylactic reactions and even death [4–7]. Anaphylaxis may be the result of a variety of triggers. Several case reports described anaphylaxis following regional or general anaesthesia as the first presentation of mastocytosis [8, 9]. The diagnosis may be difficult as anaphylaxis may develop with a delay of hours [10]. Furthermore, drugs that are known potential mast cell elicitors are frequently used pharmaceuticals in the ICU [9]. Physicians should therefore realise that adjustment of management to avoid trigger application is necessary in order to prevent further endangerment of vital functions. However, owing to the low but probably under-reported prevalence of the disease, most critical care physicians are not familiar with the management of the patient with mastocytosis. In the following sections, we review the pathogenesis and prophylactic measures of mast cell mediator release, with an emphasis on critical care management.

**Table 1:** Effects of mast cell mediators in mastocytosis

Mediator(s)	Features
Cardiovascular	
Prostaglandins	Flushing (increased heart rate and increased cardiac output)
Protease (Chymase)	Increased blood pressure
Histamine	Increased vasopermeability
Histamine, prostaglandin D_2_, leukotrienes, and platelet-activating factor	Vasodilatation and hypotension
Platelet-activating factor	Arrhythmia
Cutaneous	
Histamine, prostaglandin D_2_, and platelet-activating factor	Urticaria with or without angioedema
Histamine	Pruritus
Respiratory	
Histamine, leukotrienes, prostaglandin D_2_, and platelet-activating factor	Bronchusconstriction
Prostaglandin D_2_ and leukotrienes	Increased mucus production
Platelet-activating factor and leukotrienes	Pulmonary oedema
Histamine	Rhinitis
Gastrointestinal	
Histamine	Increased gastric acid secretion
Histamine	Diarrhoea
Platelet-activating factor	Abdominal ache
Hematologic	
Heparin and proteases	Coagulation disturbances
Remainder	
Histamine	Headache
Heparin, interleukin-6 (IL-6), and tryptase	Osteopenia and osteoporosis
Pro-inflammatory cytokines (for example, tumour necrosis factor-alpha) and chemokines	Fatigue, weight loss, local inflammation, oedema formation, and leukocyte migration
Growth factor (IL-6)	Fever
Tryptase	Endothelial activation with consecutive inflammatory reactions

## Mastocytosis

### Incidence and prevalence

The incidence of mastocytosis has been estimated to be between 1:1,000 and 2:100,000, but several cases are not recognized as caused by mastocytosis [2, 11]. The prevalence of mastocytosis was recently studied in the adult population of the Groningen region in The Netherlands and appeared to be at least 13 cases per 100,000 inhabitants [12].

### Pathophysiology

**Fig. 1:**
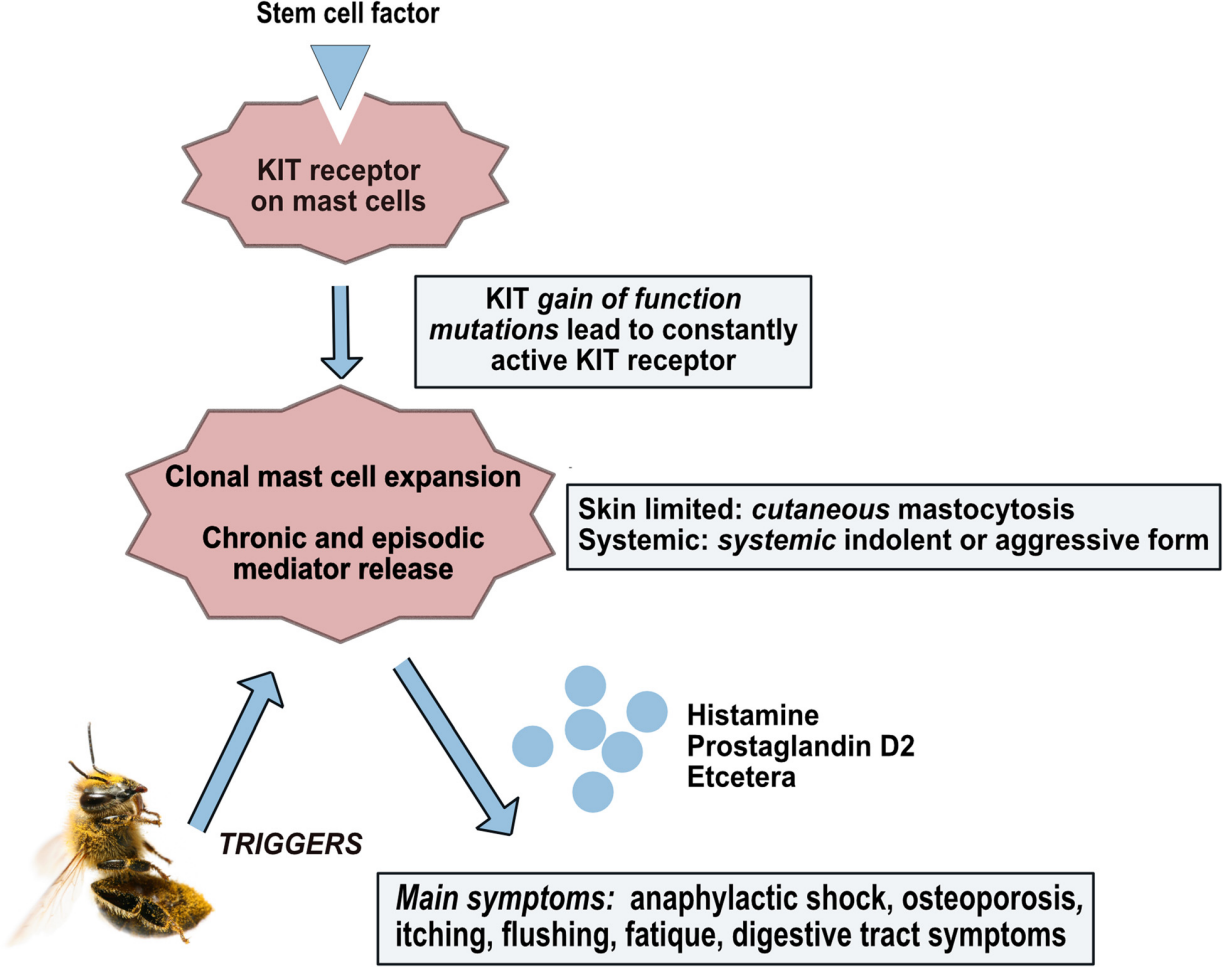
Simplified pathophysiology of mastocytosis. The vast majority of patients with mastocytosis have a gain-of-function mutation in the KIT-receptor gene. KIT encodes for a tyrosine kinase which functions as a receptor (CD117) for the stem cell factor (formally known as mast cell growth factor). The mutation results in continuous activation and stimulation even without binding with the mast cell growth factor

Mast cells develop from pluripotent stem cells in the bone marrow and spleen. In mastocytosis, the majority of cases are associated with gain-of-function point mutations of KIT, a tyrosine kinase receptor located on mast cells. As a consequence, the receptor is continuously activated, leading to uncontrolled growth and activation of mast cells (Fig. 1) [2]. Mast cell progenitors are the only myeloid cells that exit the bone marrow before complete maturation. The destination of mast cells is beneath or in the epithelium, close to vessels, smooth muscle cells, and glandular tissue, where they act as outposts of the immune system against exogenous allergens and pathogens [2]. Subjects with mastocytosis can experience non-specific symptoms like generalized pruritus, flushing, diarrhoea, abdominal pain, anaphylaxis, and osteoporosis due to a release of mast cell mediators, weight loss, malabsorbtion, cytopenia, organomegaly, and bone pain and fracture due to infiltration of mast cells in organs in advanced or aggressive mastocytosis [1].

### Diagnosis and classification

Mastocytosis is classified by the World Health Organization (WHO) in the category of ‘myeloproliferative neoplasms’ and can be subdivided into cutaneous and systemic mastocytosis and mast cell neoplasm (Table 2). To establish the diagnosis of systemic mastocytosis, either one major and two minor or three minor WHO criteria have to be fulfilled (Table 3). The indolent form is the most prevalent subtype of systemic mastocytosis [13]. An increased basal level of the mast cell mediator tryptase (more than 20 ng/mL) in serum may indicate the presence of the disease [14]. When mastocytosis is suspected (for example, in a patient presenting with anaphylactic shock due to a wasp sting), a marked increased tryptase concentration is the hallmark of the diagnosis [1]. One can also expect to find coagulation disturbances, caused by the mast cell mediators heparin and protease, and (methyl)histamine in urine. In this particular example, immunoglobulin (Ig) against wasp-antigen (rVes v5) shall be positive, revealing an IgE-mediated reaction [1, 13, 14].

**Table 2:** **World Health Organization classification of mastocytosis**

Variant	Subvariants
1. Cutaneous mastocytosis	
	- Urticaria pigmentosa
	- Diffuse cutaneous mastocytosis
	- Mastocytoma of skin
2. Systemic mastocytosis	
Indolent systemic mastocytosis	- Smouldering systemic mastocytosis Isolated bone marrow mastocytosis
Aggressive systemic mastocytosis	- Lymphadenopathic systemic mastocytosis with eosinophilia
Systemic mastocytosis with an associated non-mast cell lineage disorder	- Systemic mastocytosis with myelodysplastic syndrome
	- Systemic mastocytosis with myeloproliferative disorder
	- Systemic mastocytosis with chronic myelomonocytic leukaemia
	- Systemic mastocytosis with non-Hodgkin’s lymphoma
	- Systemic mastocytosis with hypereosinophilic syndrome
Mast cell leukaemia	- Aleukaemic mast cell leukaemia
3. Mast cell neoplasms	
Mast cell sarcoma	
Extracutaneous mastocytoma

**Table 3:** World Health Organization criteria for systemic mastocytosis

Major criterion
- The existence of 15 or more multifocal mast cell clusters in the bone marrow or in other tissue biopsies
Minor criteria
- Basal tryptase level of more than 20 ng/mL
- Either (a) more than 25% of mast cells in infiltrates of bone marrow or other extracutaneous organs are atypical or spindle-shaped or (b) more than 25% of mast cells in bone marrow aspirate are immature or atypical.
- Co-expression of CD117 with CD25 or CD2 (or both) on mast cells
- Codon 816 mutation in the C-KIT gene

To establish the diagnosis of systemic mastocytosis, either one major and two minor or three minor World Health Organization criteria have to be fulfilled. Patients who have a history of mast cell activation symptoms without skin lesions but who do not fully meet diagnostic criteria for systemic mastocytosis are classified as having mast cell activation syndrome [9, 17]

### Anaphylaxis and risk factors

Stimulation of mediator release occurs by either IgE- or non-IgE-mediated mechanisms, including physical stimuli and drugs. The marked increased mediator release from the elevated mast cell numbers explains the much higher incidence as well as severity of anaphylaxis in mastocytosis patients as compared with the general population [3]. Symptoms appear to be more severe in patients with extensive systemic disease, which is reflected by a higher serum tryptase level [15]. A study that described the trigger factors for anaphylaxis in patients with mastocytosis found that the major precipitating agents were Hymenoptera (wasps, bees, ants, and flies) stings (27 %), foods (24 %), and medications (18 %) [15]. The prevalence of Hymenoptera venom allergy among patients with mastocytosis varies between 23 % and 47 % [11, 12]. Hymenoptera stings mostly cause severe anaphylactic reactions (grade III), whereas foods and medication lead to milder systemic reactions (grade II). However, fatal cases of drug hypersensitivity have been described in the literature [7, 8]. Medications that are potential initiators of mastocyte degranulation on the basis of case reports and drugs associated with histamine release in general are listed in Table 4.

**Table 4:** Medications which are known potential triggers of mast cell degranulation

	Opioids	Hypnotics	Muscle relaxants	(Local) anaesthesia	Volatile anaesthetics
Avoid			Mivacurium	Nefopam	
			Atracurium		
Avoid rapid perfusion	Morphine Codeine	Thiopental	Succinylcholine Rocuronium	Lidocain Bupivacain	
(if possible, use alternative agents)					
Low risk	Fentanyl Sufentanil Remifentanil Alfentanil	Midazolam Propofol Etomidate Ketamine	Cis-atracurium Pancuronium Vecuronium	Ropivacain	Desfurane Sevoflurane Enflurane Isoflurane
				Paracetamol	

### Critical care management

Anaphylactic shock and complications are related to mast cell degranulation; that is, spontaneous bleeding may present features of mastocytosis [6, 16]. When present, shock needs to be treated with the usual treatment, including the administration of epinephrine. For all patients with mastocytosis, epinephrine should be readily available as it hinders mast cell degranulation and is the first drug of choice during haemodynamic failure in these patients [7]. Haemorrhage with disturbed coagulation tests can be treated with protamin and plasma coagulation factors to reverse the anticoagulant effects of heparin [9].

It is important to realise that, during further ICU treatment, patients with mastocytosis are inevitably exposed to triggers of mast cell degranulation, which may be followed by severe anaphylactic responses [10]. When a patient with known mastocytosis is admitted to the ICU, it may be helpful to make a risk profile to estimate the chance of developing anaphylactoid reactions. The risk profile includes the form of mastocytosis, the basal serum tryptase level as a marker of the total mast cell load, previous anaphylactic reactions, and present mast cell activation status comprising recent reactions to triggers. Histamine-releasing drugs, such as opioids and neuromuscular blocking agents, should be avoided (Table 4). Additionally, infusion of cold solutions, trauma, friction, and other mechanical factors are elicitors of mast cell degranulation and should be prevented. Also, several antibiotics and radio contrast materials are known activators of mast cells [9]. Even anxiety may trigger mast cell degranulation, for which benzodiazepines may be considered. Continuous infusion of medication and solutions theoretically leads to less release of mast cell mediators and is preferred over single boluses.

When (surgical) intervention is planned during ICU admission, precautions must be taken. The anaesthetist should be conscious of the potential triggers and consequences of mast cell degranulation and be informed about the risk profile of the patient. Pre-medication may include benzodiazepines, H1- and H2-receptor antagonists to block the cascade of mast cell mediators, and glucocorticoids as anti-inflammatory and mast cell stabilizers. Because mast cell degranulation has been observed hours after trigger application, we recommend continuing medication for at least 4 hours after trigger application. Non-steroidal anti-inflammatory drugs (NSAIDs) are effective prophylactic drugs because of their antiprostaglandin actions. However, they should be given only to patients who have received these drugs previously, as NSAIDs are also associated with serious anaphylactoid reactions [9].

## Conclusions

Mastocytosis is a clonal disorder characterized by the proliferation and accumulation of mast cells in different tissues. After trigger application, an overwhelming release of mast cell mediators may lead to severe haemodynamic compromise and multi-organ failure. Knowledge of the pathophysiology of the disease is of paramount importance for adequate treatment and the prevention of secondary events of mast cell degranulation due to mechanical or pharmacological triggers. During treatment, physicians should avoid potential triggers of mast cell degranulation, consider giving pre-medication before interventions, and be aware of and prepared for the clinical manifestations caused by mast cell mediators. Avoiding medication and physical stimuli that trigger histamine release and blocking the cascade of actions of mast cells constitute the mainstay of therapy besides treatment of anaphylaxis.

## Abbreviations

Ig: Immunoglobulin
NSAID: Non-steroidal anti-inflammatory drug
WHO: World Health Organization
